# A New Boron–Rhodamine-Containing Carboxylic Acid as a Sugar Chemosensor

**DOI:** 10.3390/s23031528

**Published:** 2023-01-30

**Authors:** Yuta Komori, Shun Sugimoto, Toranosuke Sato, Honoka Okawara, Ryo Watanabe, Yuki Takano, Satoshi Kitaoka, Yuya Egawa

**Affiliations:** Faculty of Pharmacy and Pharmaceutical Sciences, Josai University, 1-1 Keyakidai, Sakado 350-0295, Saitama, Japan

**Keywords:** borinic acid, borinate, boron, rhodamine, sensor, glucose, diabetes

## Abstract

We propose a boron–rhodamine-containing carboxylic acid (BRhoC) substance as a new sugar chemosensor. BRhoC was obtained by the Friedel–Crafts reaction of 4-formylbenzoic acid and *N,N*-dimethylphenylboronic acid, followed by chloranil oxidation. In an aqueous buffer solution at pH 7.4, BRhoC exhibited an absorption maximum (Abs_max_) at 621 nm. Its molar absorption coefficient at Abs_max_ was calculated to be 1.4 × 10^5^ M^−1^ cm^−1^, and it exhibited an emission maximum (Em_max_) at 644 nm for the excitation at 621 nm. The quantum yield of BRhoC in CH_3_OH was calculated to be 0.16. The borinate group of BRhoC reacted with a diol moiety of sugar to form a cyclic ester, which induced a change in the absorbance and fluorescence spectra. An increase in the D-fructose (Fru) concentration resulted in the red shift of the Abs_max_ (621 nm without sugar and 637 nm with 100 mM Fru) and Em_max_ (644 nm without sugar and 658 nm with 100 mM Fru) peaks. From the curve fitting of the plots of the fluorescence intensity ratio at 644 nm and 658 nm, the binding constants (*K*) were determined to be 2.3 × 10^2^ M^−1^ and 3.1 M^−1^ for Fru and D-glucose, respectively. The sugar-binding ability and presence of a carboxyl group render BRhoC a suitable building block for the fabrication of highly advanced chemosensors.

## 1. Introduction

Boronic acid and borinic acid are organic derivatives of boric acid (B(OH)_3_), which contain three hydroxyl groups. The substitution of one of the three hydroxyl groups of B(OH)_3_ with an alkyl or aryl group yields a boronic acid, represented as RB(OH)_2_, and the substitution of two hydroxyl groups of B(OH)_3_ yields a borinic acid, represented as RR’BOH.

Many researchers have used boronic acids as a sugar-recognition moiety in chemosensors [1,2,3]. Indeed, boronic acids react with the diol moieties of sugars to form cyclic esters. This cyclic esterification reaction causes a change in the optical properties of the chemosensors. Many boronic acid-based sugar chemosensors have been developed using sophisticated designs [4,5], with two of them being approved for clinical use [6,7].

An interesting property of boronic acid-based sugar chemosensors is their ability to bind to sugar in aqueous solutions. Although sugar receptors without boronic acid have been developed, many of them function in organic solvents because their binding ability is weakened in polar solvents [8,9,10]. Furthermore, sugar receptors need to combine with effective signaling functions for sugar. In contrast, the boronic acid of chemosensors can function not only as a sugar receptor but also as a trigger for signaling mechanisms. The change in the structure of the boronic acid component through sugar binding induces optical and electrochemical changes, which are suitable for signaling in chemosensing applications [11,12]. Thus, boronic acid is the most widely used motif as a sugar chemosensor because it functions well in aqueous solutions and generates signals.

Although less common than boronic acid-based chemosensors, borinic acid-based chemosensors have also been developed [13,14,15]. The fact that there are fewer borinic acid-based chemosensors than boronic acid-based chemosensors can be attributed to the limited availability of borinic acids as reagents. Moreover, designing chemosensors comprising a borinic acid moiety that provide an optical response in the presence of target analytes is a difficult task.

**Scheme 1:**
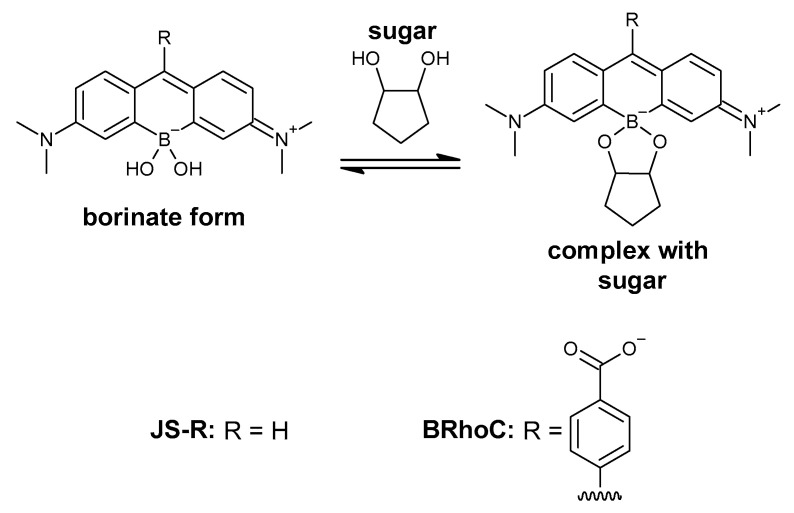
Sugar reactivities of compounds JS-R and BRhoC.

Despite the design challenges, our team synthesized a sugar chemosensor containing borinic acid and named it JoSai-Red (JS-R) after Josai University, Saitama University, and the fluorescent color of the chemosensor (Scheme 1) [13]. The structure of JS-R is similar to that of pyronin Y, which is a xanthene fluorescent dye. Because the borinic acid group of JS-R is characterized by Lewis acidity, the boron center accepts a hydroxide anion to form an anionic borinate (see Scheme 1). Given its low p*K*_a_ value (4.0), JS-R is found in borinate form in neutral solutions. Notably, the borinate form of JS-R interacts with sugars, causing changes in the absorbance and fluorescence spectra of the chemosensor. Indeed, these optical changes result from the presence of the borinate moiety, which directly affects the characteristics of the fluorophore. Although a few reports have been published on the interaction between borinate compounds and sugars, no reports exist of the successful conversion of the interaction of the borinate group with sugars into a fluorescence change, apart from the studies focusing on dyes characterized by the JS-R skeleton. A borinate H_2_O_2_ chemosensor named Rachael Fluor_620_, which contains the JS-R skeleton and was independently synthesized by the Stains group, also exhibits sugar responsiveness [14]. Indeed, JS-R is expected to represent a novel type of sugar chemosensor; however, a few problems are associated with its synthesis. Although JS-R is synthesized in a single step using a Friedel–Crafts reaction, the yield of the target compound was 0.86% [13], which needs to be improved. Furthermore, JS-R lacks the modifiable functional groups required to develop more sophisticated chemosensors.

One of the representative fluorescent dyes with a xanthene skeleton is rhodamine, which has a benzene ring at the ninth position of the xanthene skeleton. The substituted benzene ring enables various chemical modifications, and many kinds of chemosensors based on the rhodamine skeleton have been developed [16,17,18,19]. Furthermore, recent reports have demonstrated interesting features of fluorescent dyes in which the oxygen atom of rhodamine is replaced with heteroatoms [20,21,22,23,24], such as silicon [25,26,27,28,29,30], phosphorus [31,32,33,34], sulfur [35,36], and boron [14,15]. Only two cases of boron-substituted rhodamine have been reported and there is still much room for further investigation.

In this study, we report the development of a new sugar chemosensor containing a borinate moiety in a rhodamine-like structure. Using 4-formylbenzoic acid as the starting material, a new JS-R derivative, boron–rhodamine-containing carboxylic acid (BRhoC in Scheme 1), was obtained as the third example of boron–rhodamine compounds [14,15]. Furthermore, BRhoC has the advantage that the carboxyl group on the rhodamine skeleton can be chemically modified. Herein, we also present evidence of the potential of BRhoC as a sugar chemosensor.

## 2. Materials and Methods

### 2.1. Materials

4-formylbenzoic acid, 3-(*N,N*-dimethylamino)phenylboronic acid, acetic acid (glacial), boron trifluoride-diethyl ether complex (BF_3_·Et_2_O), and 4-(2-hydroxyethyl)-1-piperazineethanesulfonic acid (HEPES) were obtained from Sigma-Aldrich Japan (Tokyo, Japan); D-fructose (Fru) and D-glucose (Glc) were obtained from FUJIFILM Wako Pure Chemical Corp. (Osaka, Japan); chloranil was obtained from Tokyo Chemical Industry Co., Ltd. (Tokyo, Japan); and cresyl violet (fluorescence reference standard) was obtained from Cosmo Bio Co., Ltd. (Tokyo, Japan). All chemicals were used without further purification. 

### 2.2. Apparatus

Reactions were monitored with thin-layer chromatography (TLC) silica gel 60 F₂₅₄ (Merck KGaA, Darmstadt, Germany). Medium-pressure liquid chromatography was performed with a Smart Flash AI-580S instrument (Yamazen Corp., Osaka, Japan) using a silica gel cartridge column. NMR spectra were recorded using an AVANCE Neo 400 NMR spectrometer (Bruker Japan K.K., Kanagawa, Japan). Tetramethylsilane was used as the internal standard in the ^1^H and ^13^C NMR spectrometry experiments. BF_3_·Et_2_O in toluene-*d*_8_ was used as the external standard in the ^11^B NMR spectroscopy experiments. Mass spectrometry (MS) data were collected using a JMS-700(2) MStation (JEOL Ltd., Tokyo, Japan); elemental analyses were conducted using an MT-6 CHN analyzer (Yanaco Technical Science Corp., Tokyo, Japan); ultraviolet–visible (UV–Vis) absorption spectra were recorded using a V-560 spectrometer (JASCO Corp., Tokyo, Japan); and fluorescence spectra were recorded using an RF-5300PC instrument (Shimadzu Corp., Kyoto, Japan).

### 2.3. Synthesis of BRhoC

3-(*N,N*-dimethylamino)phenylboronic acid (1.00 g, 6.06 mmol) and 4-formylbenzoic acid (364 mg, 2.42 mmol) were dissolved in 5.0 mL of acetic acid (glacial) in a reaction flask that was subsequently fitted with a calcium chloride drying tube; the mixture inside it was then stirred at 85 °C for 2 h. The reaction solution was then allowed to cool to room temperature. Chloranil (1.79 g, 7.28 mmol) and acetic acid (10 mL) were added to the reaction solution, which was stirred at room temperature for 0.5 h. Acetic acid was then removed by evaporation and the obtained residue was dried in vacuo. The dried residue was dissolved in 12 mL of dichloromethane and the resulting solution was filtered using a paper filter. The filtrate was subjected to medium-pressure liquid chromatography using a silica gel cartridge column. The composition of the mobile phase was modified stepwise: CH_2_Cl_2_/CH_3_OH, 100/0 (10 min); 95/5 (10 min); 50/50 (10 min); and 0/100 (30 min). The target fractions were pooled and the solvent was removed by evaporation. Distilled water (400 mL) was then added to the residue. The pH of the aqueous solution thus obtained was adjusted to 7 by adding a small amount of 1 M NaOH (aq.); the solution was then filtered using filter paper. The pH of the filtrate was adjusted to 2 by adding 1 M HCl (aq.), prompting the formation of a precipitate, which was collected with a membrane filter, yielding a black-blue solid (142 mg and 9.4% yield, which was calculated considering the purity of the borinic acid form of BRhoC (90.1%)). ^1^H NMR (400 MHz, CD_3_OD, Figure S1): *δ* 8.18 (d, 2H), 7.39 (d, 2H), 7.31(d, 2H), 6.98 (d, 2H), 6.61 (dd, 2H), 3.29(s, 12H). ^13^C NMR (100 MHz, CD_3_OD, Figure S2): *δ* 169.52, 169.25, 156.41, 145.15, 141.05, 132.00, 130.76, 130.35, 129.95, 118.74, 113.11, 40.61. ^11^B NMR (128 MHz, CD_3_OD, Figure S3): *δ* 1.84. MS (FAB, positive mode, matrix: glycerol, solvent: CH_3_OH), Figure S4: 473. The expected *m/z* value of [BRhoC (borinate form) + glycerol − 2H_2_O + H]^+^ is 473. Elemental analysis calculated for C_24_H_24_BClN_2_O_3_ [BRhoC (borinic acid form)·Cl^−^] C 66.31%, H 5.56%, and N 6.44%; found C 63.29%, H 6.14%, and N 5.80%. Melting point: >300 °C. 

### 2.4. Spectral Measurement of the pH and Sugar Response of BRhoC

For the pH-titration experiment, BRhoC was dissolved in water containing 10 mM HEPES. The pH value was adjusted by adding a small amount of 1 M HCl or 1 M NaOH aqueous solution to the BRhoC solution, and the UV–Vis absorption and fluorescence spectra of BRhoC were recorded at different pH values. The added volume of HCl or NaOH solution was so small that the effect of BRhoC dilution in the spectra was negligible. For the sugar-response experiment, BRhoC was dissolved in a buffer solution (10 mM HEPES, pH 7.4) and the UV–Vis absorption and fluorescence spectra of the solution were recorded with varying sugar concentrations. To obtain the p*K*_a_ values and binding constants, a curve-fitting analysis was performed using the KaleidaGraph software (Version 4.01) with Equations (1) and (2) [37,38]. 

## 3. Results and Discussion

### 3.1. Synthesis of BRhoC

**Scheme 2:**
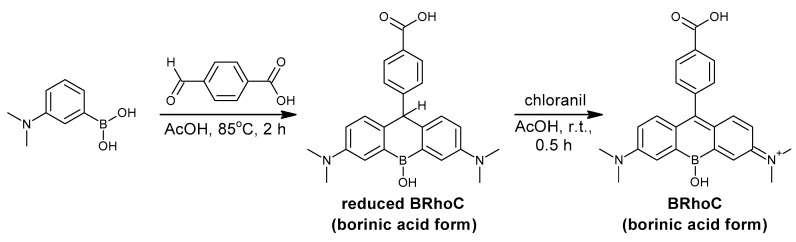
Synthesis of BRhoC.

**Figure 1:**
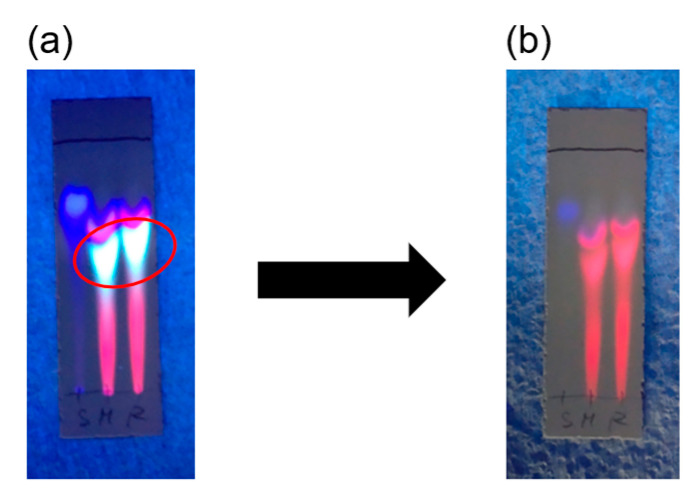
TLC images. Photos taken under irradiation with ultraviolet light at a 365 nm wavelength of a TLC plate obtained after conducting a TLC experiment of the reaction between 3-(*N,N*-dimethylamino)phenylboronic acid and 4-formylbenzoic acid (developing solvent: CH_3_OH). The photo in (**a**) is of the TLC plate immediately after development (the red circle indicates the spots due to reduced BRhoC), and the photo in (**b**) is of the same plate after 5 min exposure to the light of an ordinary fluorescent lamp.

In the previously described synthesis of JS-R [13], 3-(*N,N*-dimethylamino)phenylboronic acid and the corresponding aldehyde (4-formylbenzoic acid) were used as starting materials; however, the main product of the reaction was the reduced BRhoC and not the BRhoC (Scheme 2). The formation of the reduced BRhoC was inferred from the MS spectrum shown in Figure S5 and the results of the TLC test (Figure 1). On the TLC plate, the reduced BRhoC was observed as a light-blue spot under the irradiation of UV light at a 365 nm wavelength; however, the said spot turned red after being exposed to an ordinary fluorescent lamp for 5 min. This change is because of the extension of the conjugated system upon oxidation, which was also observed in a previously published study on a Si-substituted pyronin derivative [29].

We succeeded in synthesizing BRhoC in a one-pot reaction via the post-addition of chloranil as an oxidizer, and we developed a purification method for the borinic acid form of BRhoC. After a silica gel chromatographic procedure, the obtained solid was dispersed in distilled water and the pH of the resulting solution was adjusted to 7 by adding 1 M NaOH (aq.). BRhoC is soluble in a neutral aqueous solution due to the negative charges of the carboxylate and borinate groups of BRhoC. The pH of the aqueous solution was then adjusted to 2 by adding 1 M HCl (aq.), a process that prompted the precipitation of BRhoC in the borinic acid form as chloride. The structure of BRhoC was confirmed by NMR spectroscopy (Figures S1–S3) and fast-atom bombardment (FAB)–MS (Figure S4) data. In the elemental analysis, the found values were C 63.29%, H 6.14%, and N 5.80% (calculated for C_24_H_24_BClN_2_O_3_ [BRhoC (borinic acid form)·Cl^−^] C 66.31%, H 5.56%, and N 6.44%). The purity was calculated from the nitrogen values, assuming that no nitrogen was present in the impurities (5.80/6.44 = 90.1%). Considering the purity of the borinic acid form of BRhoC (90.1%), the synthetic yield was calculated to be 9.4%, which is 10 times larger than the synthetic yield reported for JS-R (0.86%) [13]. The found values (C 63.29%, H 6.14%, and N 5.80%) agreed within an upper threshold of 0.4% with the calculated values for a hydrate form [BRhoC (borinic acid form)·Cl^−^ + H_2_O] (C_24_H_26_N_2_BClN_2_O_4_; C, 63.67%; H, 5.79%; and N, 6.19%). According to our previous study, the precipitation of JS-R requires the addition of a large amount of NaCl to an acidic solution of JS-R [13]. In contrast, BRhoC precipitates simply as a result of the acidification of its aqueous solution. This evidence suggests that the benzoic acid moiety of BRhoC contributes to the precipitation of this compound. 

### 3.2. Optical Properties of BRhoC

**Figure 2:**
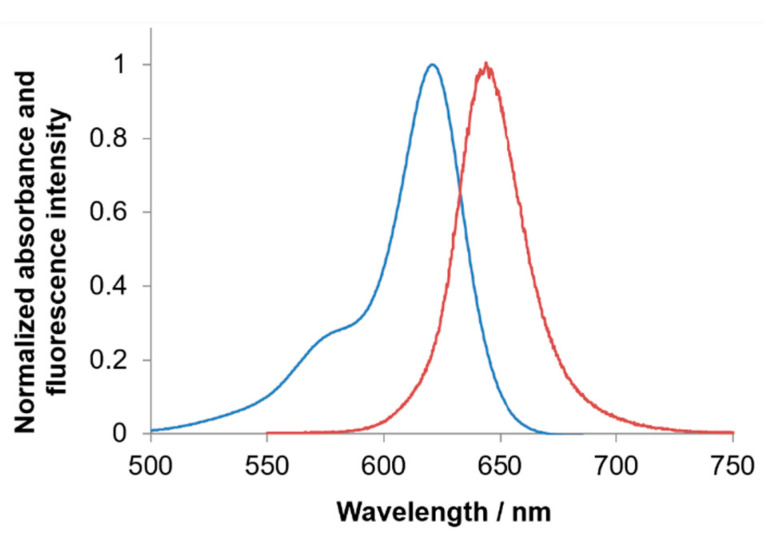
Absorption (blue line) and fluorescence (red line) spectra (λ_ex_ = 621 nm) of BRhoC (8.7 μM) in a 10 mM HEPES buffer at pH 7.4.

The optical properties of BRhoC were investigated by conducting absorbance and fluorescence spectroscopy experiments. In a buffer solution at pH 7.4, BRhoC exhibits an absorption maximum (Abs_max_) at 621 nm and the molar absorption coefficient at Abs_max_ was calculated to be 1.4 × 10^5^ M^−1^ cm^−1^ (Figure 2). BRhoC exhibits an emission maximum (Em_max_) at 644 nm when excited at 621 nm (Figure 2). These wavelengths are about 10 nm longer than those observed for JS-R (Abs_max_ = 611 nm; Em_max_ = 631 nm) and much higher than those of pyronin Y (Abs_max_ = 552 nm; Em_max_ = 569 nm, in CH_2_Cl_2_) [25]. By using cresyl violet as a reference compound, the quantum yield of BRhoC in CH_3_OH was calculated to be 0.16 [37,39]. The emission at a long wavelength and the satisfactory quantum yield demonstrate that BRhoC has application value as a fluorescent dye.

### 3.3. pH Response of BRhoC

**Figure 3:**
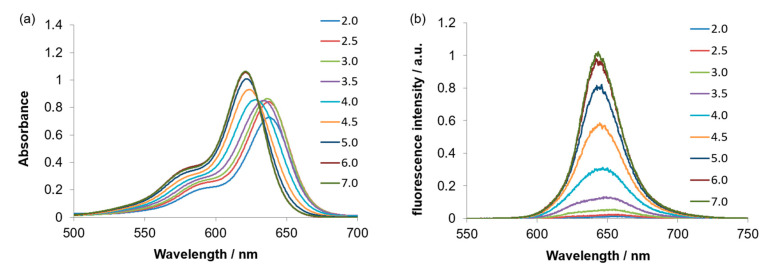
Effect of pH on the (**a**) absorption spectra, and (**b**) fluorescence spectra (excited at 621 nm) of BRhoC (8.7 μM) in a 10 mM HEPES aqueous solution (pH 2–7).

**Figure 4:**
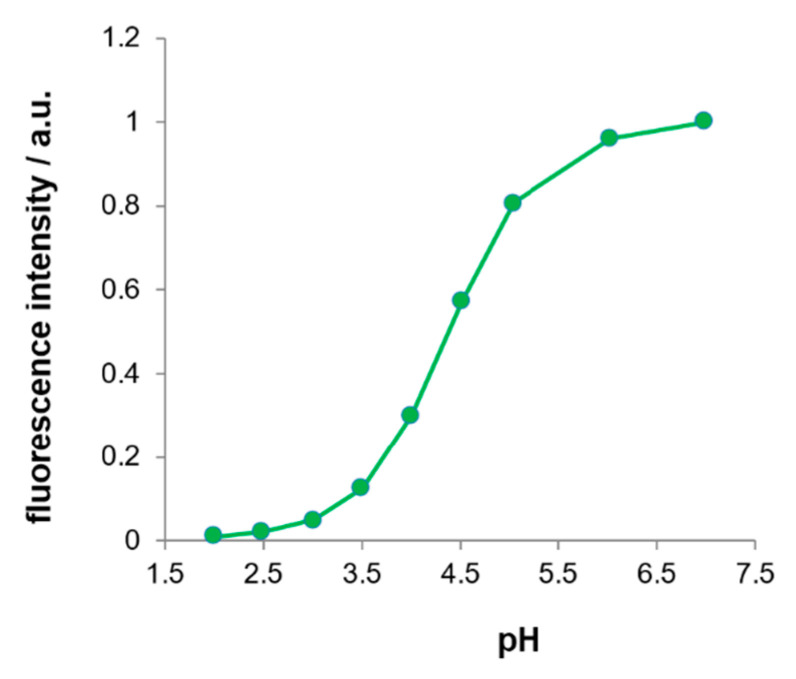
Effect of pH on the fluorescence intensity of BRhoC (8.7 μM) in a 10 mM HEPES aqueous solution.

The pH response of BRhoC was investigated by adding small amounts of HCl and NaOH to the solution of BRhoC (10 mM HEPES; Figure 3 and Figure S6). The absorption spectra changed with the pH in an acidic region (Figure 3a), whereas they remained almost unchanged in an alkaline region (Figure S6a). The lack of isosbestic points in the acidic region suggests the presence of more than two chemical species [40,41]. This observation probably stems from the fact that changes in both the borinic acid and carboxylic acid moieties affect the absorbance spectrum. By contrast, the fluorescence spectrum exhibited an intensity increase as the pH increased (Figure 3b and Figure 4). This outcome implies that only the borinic acid group, not the carboxylic acid group, affects the fluorescence intensity because the borinic acid is incorporated into the fluorescent xanthene skeleton. To determine the p*K*_a_ of the borinic acid group of BRhoC, a curve-fitting analysis was conducted on the plot of the fluorescence intensity at 644 nm versus the pH (Figure 4) based on Equation (1) [37]:

*F* = *F*_0_ + (*F*_lim_ − *F*_0_)/(1 + [H^+^]/*K*_a_) (1)

where *F* is the fluorescence intensity measured at a particular pH value, *F*_0_ is the fluorescence intensity measured for the borinic acid form of BRhoC, *F*_lim_ is the fluorescence intensity measured for the borinate form of BRhoC, [H^+^] is the proton concentration, and *K*_a_ is the acid dissociation constant of the borinic acid moiety of BRhoC. The p*K*_a_ of BRhoC can thus be estimated to have a value of 4.4, which is similar to the value previously reported for the p*K*_a_ value of BRhoC of JS-R (p*K*_a_ = 4.0) [13]. 

### 3.4. Response of BRhoC to Sugar

**Figure 5:**
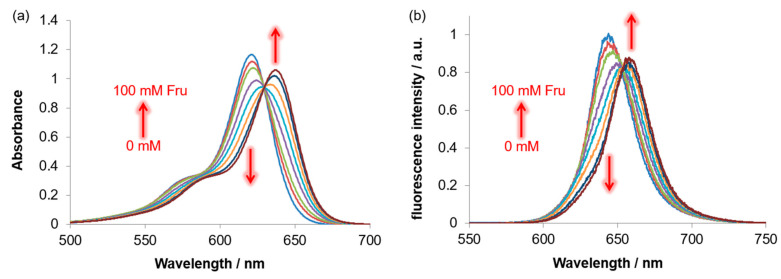
Effect of different concentrations of Fru (0, 1, 2, 5, 10, 20, 50, and 100 mM) on the (**a**) absorption spectra, and (**b**) fluorescence spectra (excited at 621 nm) of BRhoC (8.5 μM) in a 10 mM HEPES buffer at pH 7.4.

**Figure 6:**
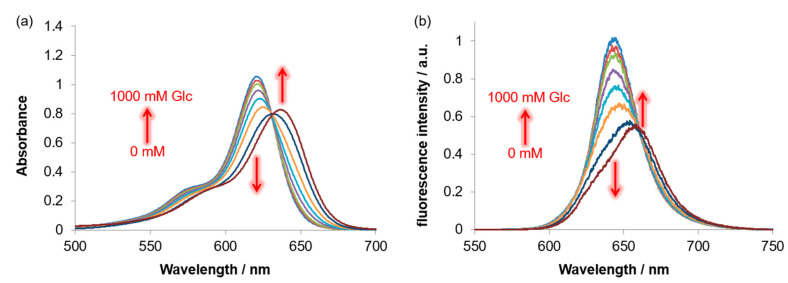
Effect of different concentrations of Glc (0, 10, 20, 50, 100, 200, 500, and 1000 mM) on the (**a**) absorption spectra, and (**b**) fluorescence spectra (excited at 621 nm) of BRhoC (8.5 μM) in a 10 mM HEPES buffer at pH 7.4.

Herein, the potential of BRhoC as a sugar chemosensor is evaluated. Figure 5 shows data that reflect the effects of Fru on the absorption and fluorescence spectra of BRhoC in a buffer solution at pH 7.4. An increase in the Fru concentration resulted in a red shift of the Abs_max_ (621 nm without Fru, 637 nm at 100 mM Fru) and Em_max_ (644 nm without Fru, 658 nm at 100 mM Fru) peaks. Similar results were obtained with Glc, although the responsiveness of BRhoC to Glc was weaker than that to Fru (Figure 6).

**Figure 7:**
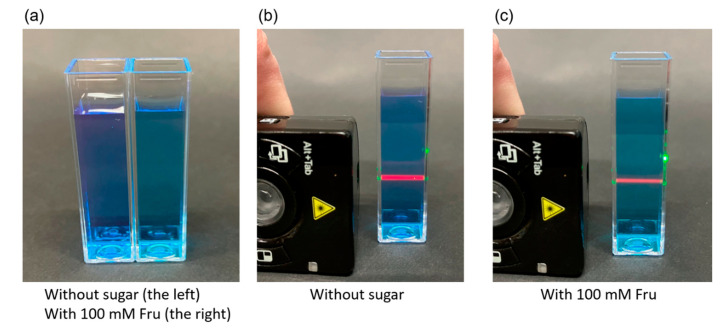
BRhoC solutions (17 μM) in a 10 mM HEPES buffer at pH 7.4. (**a**) Under an ordinary fluorescent lamp without sugar (**left**) or with 100 mM Fru (**right**). (**b**) A solution without sugar irradiated by a 532 nm laser. (**c**) A solution with 100 mM Fru irradiated by a 532 nm laser.

The sugar-induced red shift of the absorption peak indicates a decrease in the HOMO–LUMO gap, which was previously confirmed by quantum calculations using the JS-R structure [13]. Generally, the fluorescence intensity is proportional to the absorbance. This indicates that changes in the absorbance affect the fluorescence intensity. The addition of Fru reduces the absorbance at 621 nm, which is employed as the excitation wavelength. Thus, the intensity of the emitted fluorescence at an excitation wavelength of 621 nm decreased (Figure 5b). The color change upon Fru addition was observed with the naked eye (Figure 7a). When irradiated with a 532 nm laser, the solution of BRhoC emitted red fluorescence (Figure 7b) and the fluorescence intensity was reduced in the presence of Fru (Figure 7c).

**Figure 8:**
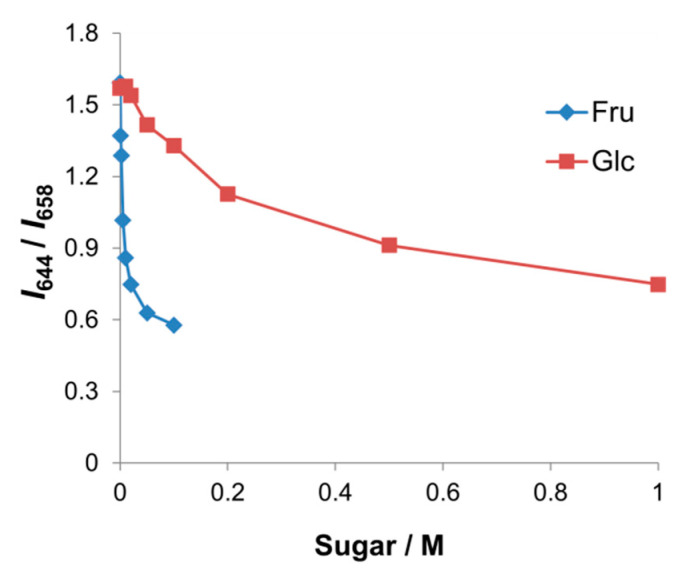
Effect of sugar on the fluorescence intensity of BRhoC (8.5 μM) in a 10 mM HEPES buffer at pH 7.4. The fluorescence intensity of BRhoC was monitored at 644 nm when excited at 621 nm.

The binding constant (*K*) of the BRhoC–sugar complexes can be calculated using a curve-fitting analysis based on Equation (2) for the sugar-response curve (Figure 8) [16]:

*FR* = (*FR*_0_ + *FR*_lim_
*K* [sugar])/(1 + *K* [sugar]) (2)

where *FR* is the fluorescence intensity ratio at 644 nm and 658 nm (*I*_644_/*I*_658_) for a particular concentration of sugar, *FR*_0_ is the initial fluorescence ratio, *FR*_lim_ is the limiting (final) fluorescence ratio, and [sugar] is the concentration of sugar. The binding constants (*K*) were determined to be 2.3 × 10^2^ M^−1^ and 3.1 M^−1^ for Fru and Glc, respectively. The dissociation constants (*K*_d_), which are the reciprocal of *K*, were calculated to be 4.3 mM and 0.32 M for Fru and Glc, respectively. The sugar-binding ability and selectivity of BRhoC are comparable to those exhibited by JS-R (*K* = 1.2 × 10^2^ M^−1^ for Fru, *K* = 3.3 M^−1^ for Glc) [13], indicating that BRhoC has the potential to be used as a sugar chemosensor.

## 4. Conclusions

We have succeeded in synthesizing a new JS-R derivative, BRhoC, and demonstrated that it works as an absorbance- and fluorescence-based sugar chemosensor. Unlike JS-R, chloranil is required for the synthesis of BRhoC. The improved synthetic yield of BRhoC compared to that of JS-R increases the likelihood of its further usage. Additionally, BRhoC has the advantage that the carboxylic acid moiety on the rhodamine-like structure can be used to chemically modify the compound. In the future, BRhoC will be used as a building block to create more advanced chemosensors for a wide range of applications.

## Data Availability

Not applicable.

## Supplementary Materials

The following supporting information can be downloaded at: https://www.mdpi.com/article/10.3390/s23031528/s1, Figure S1: ^1^H NMR spectrum of BRhoC; Figure S2: ^13^C NMR spectrum of BRhoC; Figure S3: ^11^B NMR spectrum of BRhoC; Figure S4: Mass spectrum of BRhoC; Figure S5: Mass spectrum of reduced BRhoC; Figure S6: The effect of pH on the absorption (a) and fluorescence (b) spectra of BRhoC (pH 8–13).

## References

[1] Sun X., Zhai W., Fossey J.S., James T.D. (2016). Boronic acids for fluorescence imaging of carbohydrates. Chem. Commun..

[2] Sun X., Chapin B.M., Metola P., Collins B., Wang B., James T.D., Anslyn E.V. (2019). The mechanisms of boronate ester formation and fluorescent turn-on in *ortho*-aminomethylphenylboronic acids. Nat. Chem..

[3] Williams G.T., Kedge J.L., Fossey J.S. (2021). Molecular boronic acid-based saccharide sensors. ACS Sens..

[4] Tsuchido Y., Fujiwara S., Hashimoto T., Hayashita T. (2017). Development of supramolecular saccharide sensors based on cyclodextrin complexes and self-assembling systems. Chem. Pharm. Bull..

[5] Bian Z., Liu A., Li Y., Fang G., Yao Q., Zhang G., Wu Z. (2020). Boronic acid sensors with double recognition sites: A review. Analyst.

[6] Mortellaro M., DeHennis A. (2014). Performance characterization of an abiotic and fluorescent-based continuous glucose monitoring system in patients with type 1 diabetes. Biosens. Bioelectron..

[7] Crane B.C., Barwell N.P., Gopal P., Gopichand M., Higgs T., James T.D., Jones C.M., Mackenzie A., Mulavisala K.P., Paterson W. (2015). The development of a continuous intravascular glucose monitoring sensor. J. Diabetes Sci. Technol..

[8] Davis A.P., Wareham R.S. (1998). A tricyclic polyamide receptor for carbohydrates in organic media. Angew. Chem.-Int. Ed..

[9] Davis A.P., Wareham R.S. (1999). Carbohydrate recognition through noncovalent interactions: A challenge for biomimetic and supramolecular chemistry. Angew. Chem.-Int. Ed..

[10] Sun X., James T.D. (2015). Glucose sensing in supramolecular chemistry. Chem. Rev..

[11] Egawa Y., Seki T., Takahashi S., Anzai J. (2011). Electrochemical and optical sugar sensors based on phenylboronic acid and its derivatives. Mater. Sci. Eng. C.

[12] Egawa Y., Miki R., Seki T. (2014). Colorimetric sugar sensing using boronic acid-substituted azobenzenes. Materials (Basel).

[13] Shimomura N., Egawa Y., Miki R., Fujihara T., Ishimaru Y., Seki T. (2016). A red fluorophore comprising a borinate-containing xanthene analogue as a polyol sensor. Org. Biomol. Chem..

[14] Zhou X., Lesiak L., Lai R., Beck J.R., Zhao J., Elowsky C.G., Li H., Stains C.I. (2017). Chemoselective alteration of fluorophore scaffolds as a strategy for the development of ratiometric chemodosimeters. Angew. Chem.-Int. Ed..

[15] Zhang M., Wang T., Lin X., Fan M., Zho Y., Li N., Cui X. (2021). Boron-substituted rhodamine for ratiometric monitoring dynamic of H_2_O_2_ and HOCl in vivo. Sens. Actuators B Chem..

[16] Mottram L.F., Forbes S., Ackley B.D., Peterson B.R. (2012). Hydrophobic analogues of rhodamine B and rhodamine 101: Potent fluorescent probes of mitochondria in living C. elegans. Beilstein J. Org. Chem..

[17] Zhang R., Yan F., Huang Y., Kong D., Ye Q., Xu J., Chen L. (2016). Rhodamine-based ratiometric fluorescent probes based on excitation energy transfer mechanisms: Construction and applications in ratiometric sensing. RSC Adv..

[18] Yang Y., Gao C.Y., Liu J., Dong D. (2016). Recent developments in rhodamine salicylidene hydrazone chemosensors. Anal. Methods.

[19] Wang Y., Wang X., Ma W., Lu R., Zhou W., Gao H. (2022). Recent developments in rhodamine-based chemosensors: A review of the years 2018–2022. Chemosensors.

[20] Koide Y., Urano Y., Hanaoka K., Terai T., Nagano T. (2011). Evolution of group 14 rhodamines as platforms for near-infrared fluorescence probes utilizing photoinduced electron transfer. ACS Chem. Biol..

[21] Ikeno T., Nagano T., Hanaoka K. (2017). Silicon-substituted xanthene dyes and their unique photophysical properties for fluorescent probes. Chem. Asian J..

[22] Deng F., Xu Z. (2018). Heteroatom-substituted rhodamine dyes: Structure and spectroscopic properties. Chin. Chem. Lett..

[23] Brøndsted F., Stains C.I. (2022). Heteroatom-substituted xanthene fluorophores enter the shortwave-infrared region. Photochem. Photobiol..

[24] Khan Z., Sekar N. (2022). Far-red to NIR emitting xanthene-based fluorophores. Dye. Pigment..

[25] Fu M., Xiao Y., Qian X., Zhao D., Xu Y. (2008). A design concept of long-wavelength fluorescent analogs of rhodamine dyes: Replacement of oxygen with silicon atom. Chem. Commun..

[26] Wang T., Zhao Q.J., Hu H.G., Yu S.C., Liu X., Liu L., Wu Q.Y. (2012). Spirolactonized Si-rhodamine: A novel NIR fluorophore utilized as a platform to construct Si-rhodamine-based probes. Chem. Commun..

[27] Kim E., Yang K.S., Giedt R.J., Weissleder R. (2014). Red Si–rhodamine drug conjugates enable imaging in GFP cells. Chem. Commun..

[28] Kushida Y., Nagano T., Hanaoka K. (2015). Silicon-substituted xanthene dyes and their applications in bioimaging. Analyst.

[29] Nie H., Qiao L., Yang W., Guo B., Xin F., Jing J., Zhang X. (2016). UV-assisted synthesis of long-wavelength Si-pyronine fluorescent dyes for real-time and dynamic imaging of glutathione fluctuation in living cells. J. Mater. Chem. B.

[30] Grimm J.B., Brown T.A., Tkachuk A.N., Lavis L.D. (2017). General synthetic method for Si-fluoresceins and Si-rhodamines. ACS Cent. Sci..

[31] Zhou X., Lai R., Beck J.R., Li H., Stains C.I. (2016). Nebraska Red: A phosphinate-based near-infrared fluorophore scaffold for chemical biology applications. Chem. Commun..

[32] Fukazawa A., Usuba J., Adler R.A., Yamaguchi S. (2017). Synthesis of seminaphtho-phospha-fluorescein dyes based on the consecutive arylation of aryldichlorophosphines. Chem. Commun..

[33] Grzybowski M., Taki M., Senda K., Sato Y., Ariyoshi T., Okada Y., Kawakami R., Imamura T., Yamaguchi S. (2018). A highly photostable near-infrared labeling agent based on a phospha-rhodamine for long-term and deep imaging. Angew. Chem.-Int. Ed..

[34] Lin X., Fan M., Li N., Yang J., Zhu H., Chen B., Zhu J., Zhang D., Wang T., Cui X. (2022). Phosphorus-substituted rhodamines for bioimaging of the lysosomal peroxynitrite in vivo. Dye. Pigment..

[35] Liu J., Sun Y.Q., Zhang H., Shi H., Shi Y., Guo W. (2016). Sulfone-rhodamines: A new class of near-infrared fluorescent dyes for bioimaging. ACS Appl. Mater. Interfaces.

[36] Raliski B.K., Mun D.M., Miller E.W. (2022). Sulfone rhodamines for voltage imaging. Chem.-Asian J..

[37] (2003). “Seitai-kinou Kanren Kagaku Jikken-hou”.

[38] Ward C.J., Patel P., James T.D. (2002). Boronic acid appended azo dyes–colour sensors for saccharides. J. Chem. Soc. Perkin Trans. 1.

[39] Isak S.J., Eyring E.M. (1992). Fluorescence quantum yield of cresyl violet in methanol and water as a function of concentration. J. Phys. Chem..

[40] Nowicka-Jankowska T. (1971). Some properties of isosbestic points. J. Inorg. Nucl. Chem..

[41] Lotfy H.M., Saleh S.S., Hassan N.Y., Salem H. (2014). A comparative study of novel spectrophotometric methods based on isosbestic points; Application on a pharmaceutical ternary mixture. Spectrochim. Acta-Part A Mol. Biomol. Spectrosc..

