# Supplementary material for: A New Boron–Rhodamine-Containing Carboxylic Acid as a Sugar Chemosensor
Source: Sensors (Basel). 2023 Jan 30;23(3):1528. doi: 10.3390/s23031528 (PMC9921257; doi:10.3390/s23031528)
Supplement: Supplementary file 1 [file sensors-23-01528-s001.zip › sensors-2136730-supplementary.pdf]

Supplementary Materials

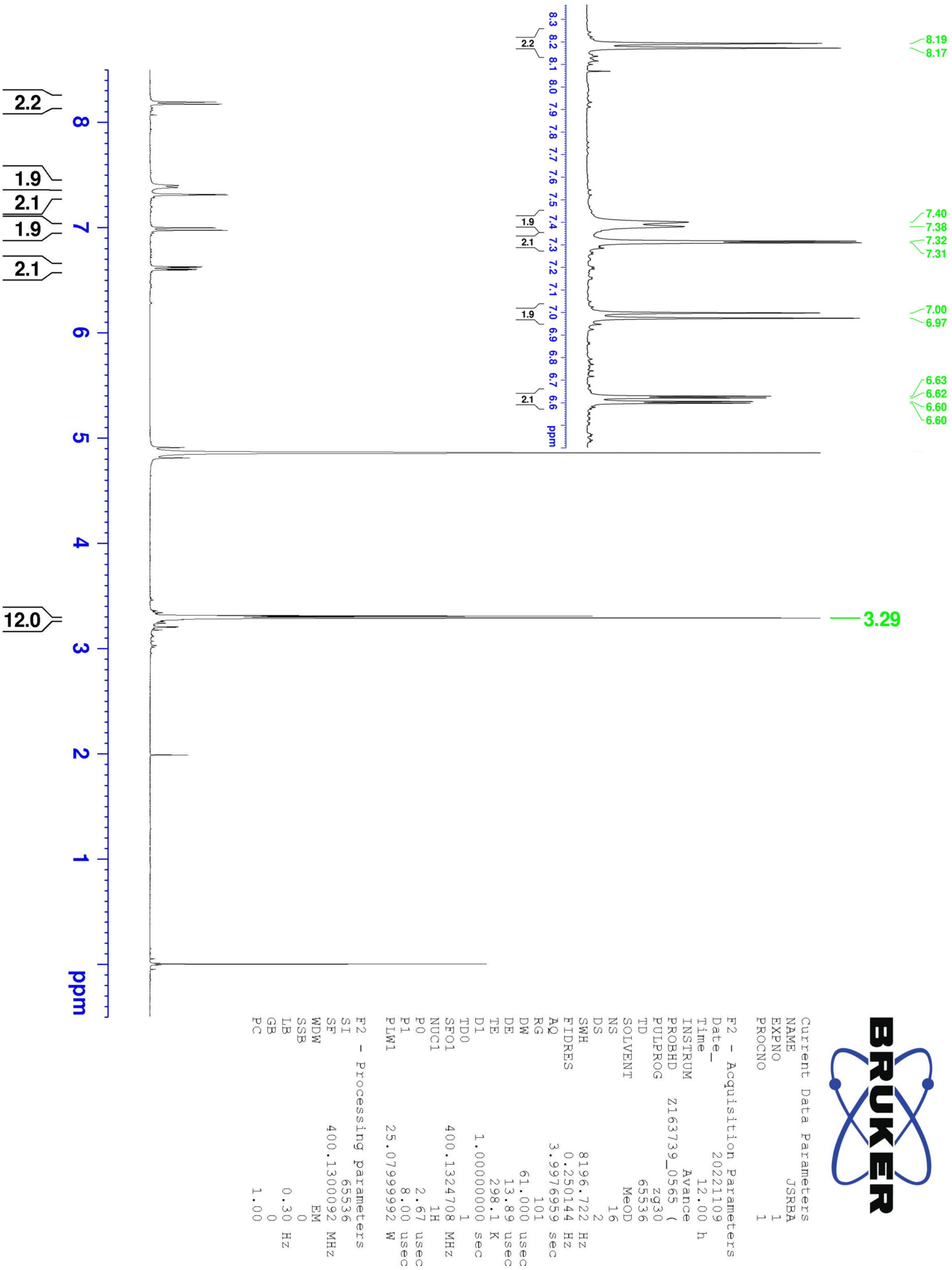

Figure S1. ^{1}H NMR spectrum of BRhoC (400 MHz, CD_3OD).

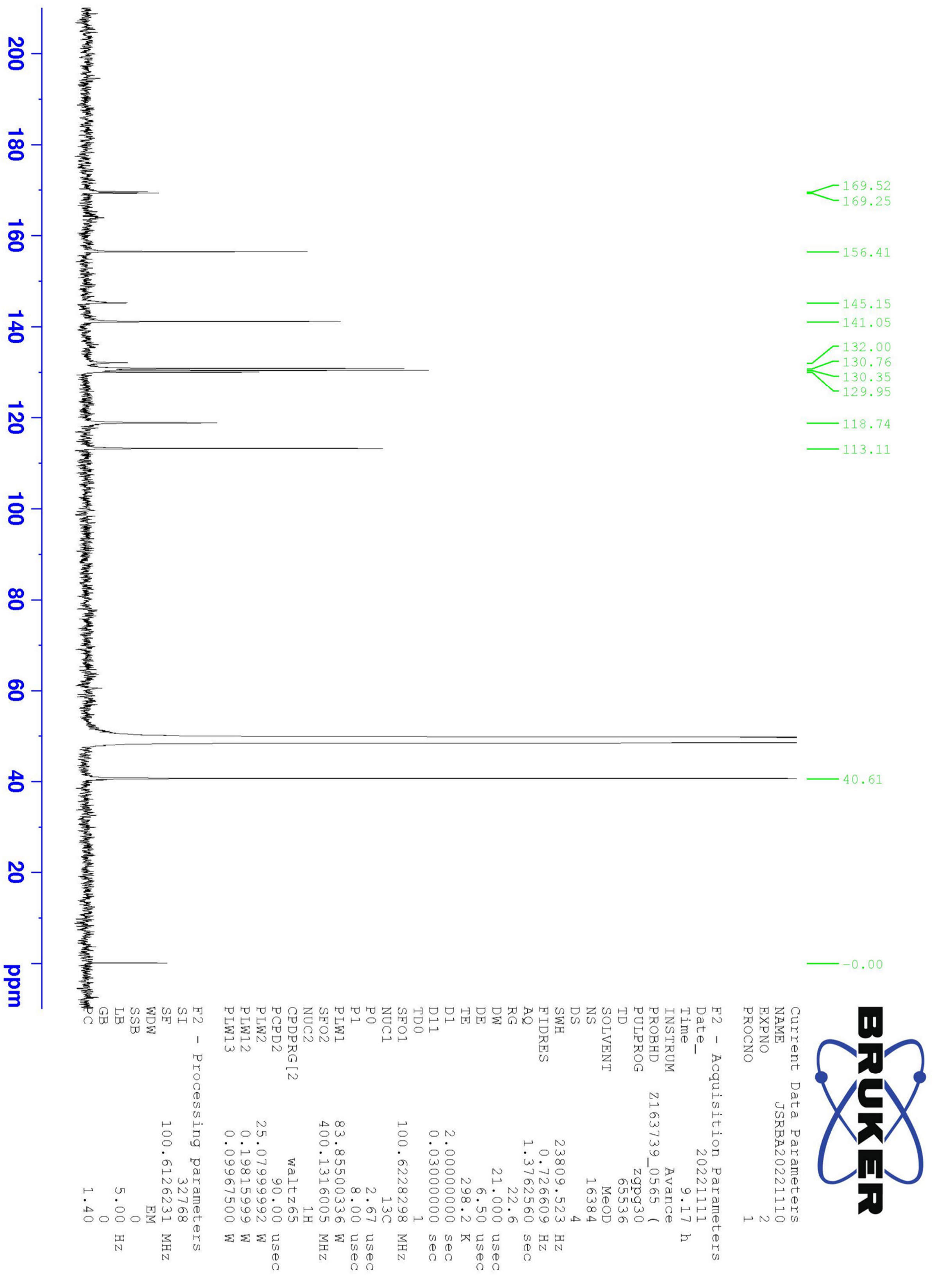

Figure S2. ^{13}C NMR spectrum of BRhoC (100 MHz, CD_3OD).

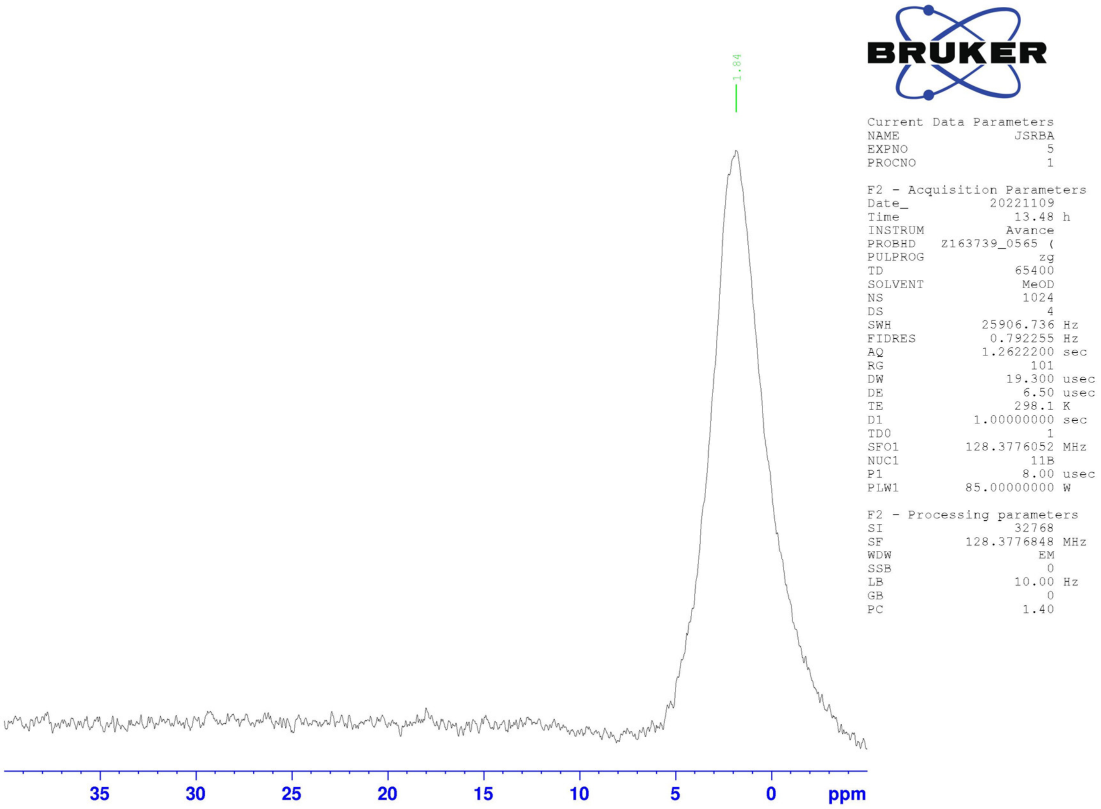

Figure S3. ^{11}B NMR spectrum of BRhoC (128 MHz, CD_3OD).

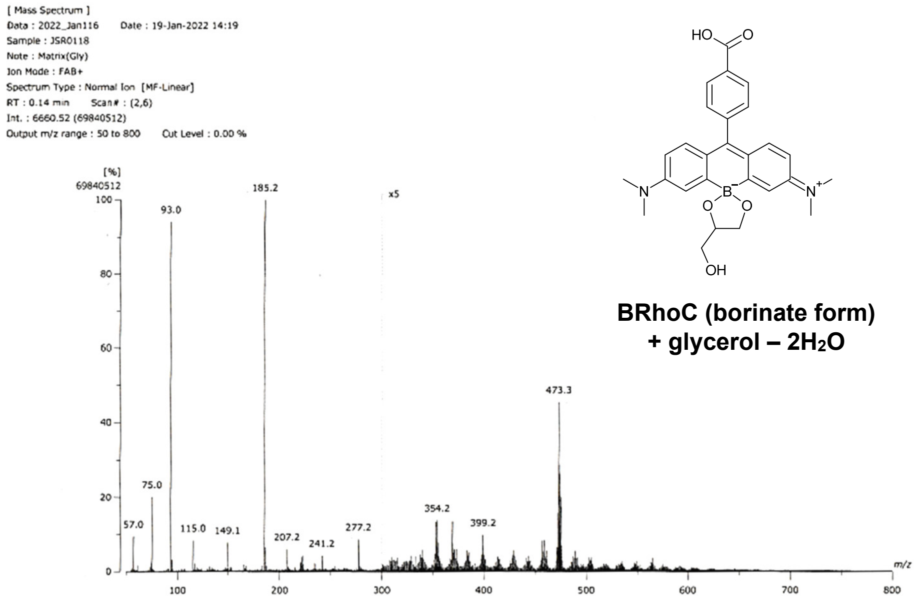

Figure S4. Mass spectrum of BRhoC (FAB, positive mode, matrix: glycerol, solvent: CH_3OH). A peak was observed at 473. The expected *m/z* value for [BRhoC (borinate form) + glycerol–$2H_2O$ + H]$^+$ is 473.

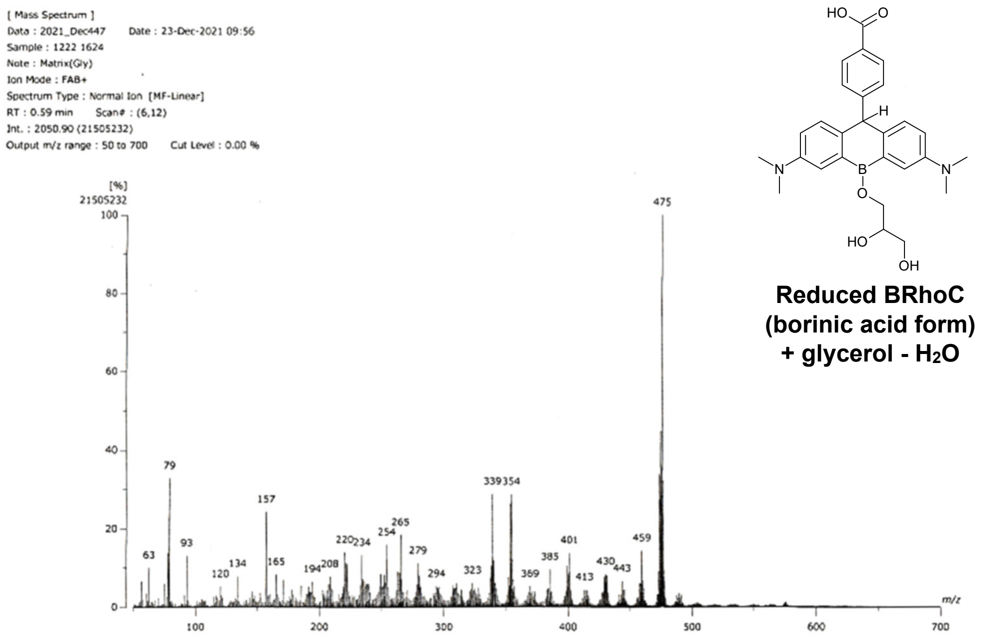

Figure S5. Mass spectrum of reduced BRhoC (FAB, positive mode, matrix: glycerol, solvent: CH_3OH). A peak was observed at 475. The expected *m*/*z* value for [the reduced BRhoC (borinic acid form) + glycerol − H_2O + H]$^+$ is 475.

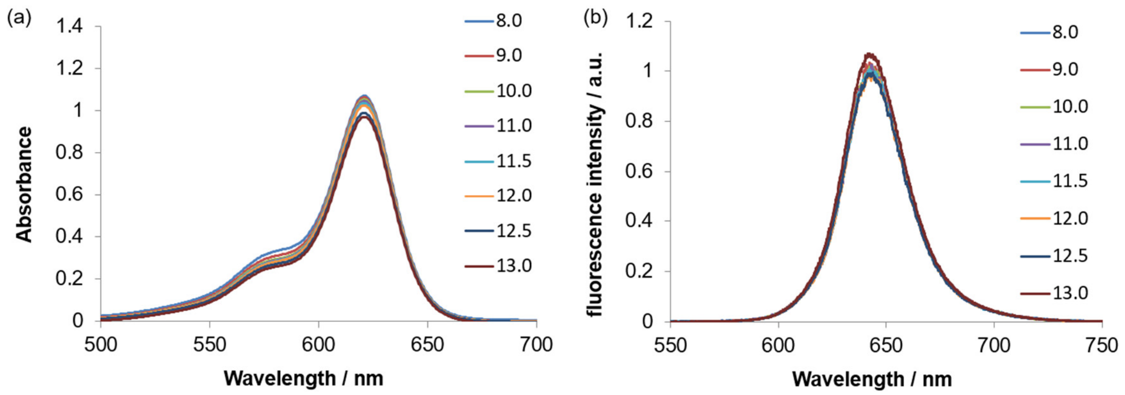

Figure S6. Effect of the pH on the absorption (**a**) and fluorescence (**b**) spectra of BRhoC (8.7 μM in 10 mM HEPES aqueous solution, pH 8–13).
